# Association between a gut microbiota-targeted dietary index and osteoarthritis/rheumatoid arthritis: a cross-sectional study

**DOI:** 10.1016/j.clinsp.2026.100995

**Published:** 2026-05-19

**Authors:** Weihua Yang, Zhi Li, Xiang Chen, Jinian Chen, Zhiming Lu

**Affiliations:** Department of Orthopedic Center, The 95 Hospital of Putian, China

**Keywords:** NHANES, Asteoarthritis, Rheumatoid arthritis, DI-GM, Cross-sectional study

## Abstract

**Background:**

Diet plays a key role in regulating the gut microbiota. This study aimed to investigate the association between the Dietary Index of the Gut Microbiome (DI-GM) ‒ a score designed to reflect the potential of a diet to support a healthy gut microbiota ‒ and different types of arthritis, an area where systematic studies remain scarce.

**Methods:**

Data from the National Health and Nutrition Examination Survey (NHANES) 2007–2018 were analyzed using multivariable logistic regression, Restricted Cubic Spline (RCS) regression, and subgroup analyses to assess associations between DI-GM and Osteoarthritis (OA) and Rheumatoid Arthritis (RA).

**Results:**

A total of 6168 participants were included, among whom 506 had Osteoarthritis (OA) and 303 had Rheumatoid Arthritis (RA). Multivariable analyses showed no significant association between DI-GM and OA (p > 0.05), but significant inverse associations with RA across models (ORs 0.74, 0.64, and 0.74; all p < 0.05). Restricted cubic spline analysis detected no significant nonlinear relationships for either outcome. Subgroup analyses suggested potential associations within certain populations.

**Conclusion:**

This cross-sectional study found an inverse association between the diet-based DI-GM score and RA, but no significant association with OA. Given the limitations of the cross-sectional design, the observed association is equally compatible with dietary changes resulting from RA diagnosis and management (reverse causality). As this study did not measure the gut microbiota, the role of the microbiome as a mediator remains speculative. Therefore, these results require confirmation in prospective studies that can account for reverse causation and directly assess the gut microbiome.

## Highlights

• Dietary gut microbiome index inversely associated with rheumatoid arthritis, not osteoarthritis.
• First study linking DI-GM to arthritis types using NHANES 2007–2018 data.
• Cross-sectional design limits causal inference; reverse causality is possible.
• Exploratory subgroup signals found in Mexican Americans and overweight adults.

## Introduction

Osteoarthritis (OA) and Rheumatoid Arthritis (RA) are the two most common forms of chronic arthritis. Characterized by synovitis, cartilage destruction, and bony changes, these conditions cause joint pain, stiffness, and deformity, resulting in substantial patient suffering and markedly reduced quality of life.^1^^,^^2^ Current estimates indicate that about 30.8 million American adults live with OA, with prevalence rising to 47.8% among older adults, and societal costs totaling hundreds of billions of dollars annually.^3^ RA, a chronic autoimmune disease that targets the joints, has a global prevalence estimated between 0.18% and 1.97%.^4^, ^5^, ^6^ Although its exact etiology is not fully understood, a Danish cohort study identified genetic factors as major contributors to risk.^7^ As leading causes of disability in middle-aged and older adults, both OA and RA often go undetected in early stages because of their insidious onset, with many cases diagnosed only after the disease has advanced.^8^ These epidemiological facts underscore the urgent need for improved prevention and earlier diagnosis.

In recent years, the Gut Microbiota (GM) has attracted considerable attention as a nexus linking multiple systemic diseases. By modulating immune homeostasis, maintaining intestinal barrier integrity, and producing bioactive metabolites such as short-chain fatty acids and bile acids, the GM plays a central role in systemic inflammation.^9^ This immunoregulatory capacity offers new perspectives on the pathogenesis of Rheumatoid Arthritis (RA). A growing body of evidence shows that RA patients exhibit gut dysbiosis, and that characteristic microbial shifts may promote the initiation and persistence of autoimmunity through mechanisms such as molecular mimicry and disruption of the intestinal barrier, the so-called “leaky gut”.^10^ By contrast, the relationship between the GM and Osteoarthritis (OA) is thought to involve low-grade systemic inflammation and metabolic dysregulation. For example, obesity driven alterations in the GM may increase circulating proinflammatory mediators such as Lipopolysaccharide (LPS), thereby indirectly intensifying joint inflammation and cartilage degradation.^11^ Thus, the GM may play distinct roles in autoimmune initiation in RA versus metabolic and inflammatory amplification in OA.

Diet is a major environmental determinant of gut microbiota composition and function.^12^, ^13^, ^14^ Unlike traditional healthy diet indices (e.g., HEI, MDS), the recently developed Dietary Index of the Gut Microbiome (DI-GM) is a diet-based scoring system intended to assess the theoretical potential of a diet to promote a gut microbiota profile associated with health.^15^, ^16^, ^17^ It is a proxy for presumed microbial effects, not a direct measure of the microbiota. Given the mechanistic differences outlined above, the authors hypothesize that a higher DI-GM score (indicating a diet with greater theoretical support for a healthy gut microbiota) may show a stronger inverse association with RA, an immune mediated disease, by systemically modulating immune function, whereas its association with osteoarthritis, which is driven primarily by mechanical wear and local metabolic factors, may be weaker or confounded by other complex factors such as obesity. To date, large-scale population studies that systematically evaluate the relationship between DI-GM and the risks of osteoarthritis and rheumatoid arthritis are lacking.

This study used data from the U.S. National Health and Nutrition Examination Survey (NHANES) 2007‒2018 to examine the association between the Dietary Index of the Gut Microbiome (DI-GM) and the risks of Osteoarthritis (OA) and Rheumatoid Arthritis (RA). The authors anticipate that the findings will clarify how this specific dietary pattern relates to arthritis and provide epidemiological data pertinent to the investigation of diet-microbiome-joint interactions, acknowledging that the microbiome component is not measured here.

## Materials and methods

### Study population

The National Health and Nutrition Examination Survey (NHANES) is a nationally representative, cross-sectional survey of the U.S. population that collects comprehensive health and nutrition data. The survey was conducted with approval from the National Center for Health Statistics Research Ethics Review Board (ERB) and in accordance with the Declaration of Helsinki. All participants provided written informed consent. NHANES datasets are publicly available on the NHANES website.

**Fig. 1:**
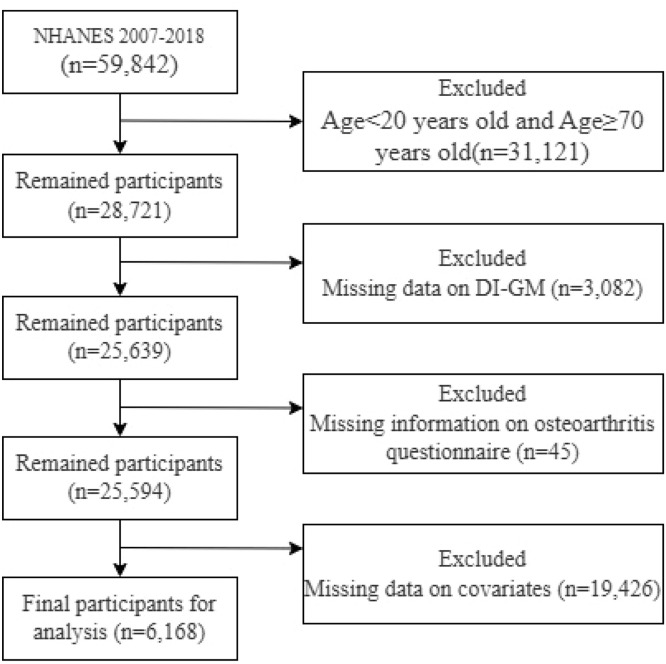
Flow chart of the participants selection process.

The authors initially identified 59,842 participants from the NHANES 2007–2018 cycles. The authors then sequentially excluded: those younger than 20-years or 70-years and older (n = 31,121); participants with missing DI-GM questionnaire data (n = 3082); participants with missing arthritis questionnaire data (n = 45); and those with missing covariate data (n = 19,426). After these exclusions, 6168 participants remained in the final analysis (Fig. 1).

### Arthritis

The definition of arthritis was based on data from the “Medical Conditions” questionnaire. In that questionnaire, participants were asked whether a doctor or other health professional had ever told them they had arthritis. Participants who answered “yes” were then asked to specify the type of arthritis: Osteoarthritis (OA), Rheumatoid Arthritis (RA), psoriatic arthritis, another type, or “unknown/refused” to classify. Based on these responses, participants were classified as having arthritis or not for this study.

### Dietary index for gut microbiota

The DI-GM was calculated from 24-hour dietary recall data in the National Health and Nutrition Examination Survey (NHANES). The index comprises 14 food or nutrient components, of which 10 are classified as beneficial and four as adverse.^16^ The beneficial components include avocados, broccoli, chickpeas, coffee, cranberries, fermented dairy products, dietary fiber, green tea, soy products, and whole grains. The adverse components comprise a high-fat diet (≥ 40% energy from fat), processed meat, red meat, and refined grains. Detailed information on the components and scoring criteria is provided in Supplementary Table S1. For beneficial components, participants received 1 point if their intake exceeded the sex-specific median, and 0 otherwise. For adverse components, 1 point was given when intake was below the sex-specific median. For high-fat diets, a fixed threshold was applied: 1 point was assigned if fat contributed less than 40% of total energy, and 0 otherwise. The overall DI-GM score was calculated by summing the scores for the 14 components. The total score ranges from 0 to 13, with higher scores indicating dietary patterns more favorable to gut microbiota health.

### Covariates

Based on prior literature and the exposure-outcome logic of this study, the authors adjusted for potential confounders known to affect arthritis. Covariates included age, sex, race, marital status (never married, married or living with a partner, widowed/divorced/separated), educational level (less than high school, high school or equivalent, more than high school), Poverty-to-Income Ratio (PIR), Body Mass Index (BMI), smoking status (never, former, current), drinking status (never, former, current), and a history of hypertension, diabetes mellitus, and cancer. Laboratory measures obtained from biospecimens included Low-Density Lipoproteins (LDL), Triglycerides (TG), Direct HDL-Cholesterol (HDL), Total Cholesterol (TC), Creatinine (CR), C-Reactive Protein (CRP), and uric acid.

The Poverty to Income Ratio (PIR) was calculated by dividing annual household income by the poverty threshold for the household size and was categorized as less than 1.3, 1.3–3.5, and greater than 3.5. According to World Health Organization criteria, Body Mass Index (BMI, kg/m^2^) was classified as < 25 (underweight or normal weight), 25 to < 30 (overweight), and ≥ 30 (obese).^18^ Hypertension was defined as an average systolic blood pressure ≥ 140 mmHg or an average diastolic blood pressure ≥ 90 mmHg, a physician diagnosis, or current use of antihypertensive medication.^19^ Diabetes mellitus was defined as an HbA1c level ≥6.5%, a fasting plasma glucose level ≥126 mg/dL, a physician diagnosis of diabetes, or current use of insulin.^20^ Cancer status was based on self‑reported, physician‑diagnosed cancer.

### Statistical analysis

The study used multivariable logistic regression to assess the association between DI-GM and arthritis, adjusting for potential confounders. Odds Ratios (ORs) were estimated using three models: Model 1 was unadjusted; Model 2 adjusted for age, sex, and race; and Model 3 was fully adjusted, adding marital status, education level, the Poverty-to-Income Ratio (PIR), Body Mass Index (BMI), smoking status, alcohol consumption, hypertension, diabetes, cancer, LDL, Triglycerides (TG), HDL, Total Cholesterol (TC), Creatinine (CR), C-Reactive Protein (CRP), and uric acid to the variables in Model 2. Restricted Cubic Spline (RCS) regression with three knots was used to evaluate potential nonlinear associations between DI-GM and arthritis. Subgroup analyses were performed by sex, age, race, marital status, education level, PIR, BMI, smoking status, alcohol consumption, hypertension, diabetes, and cancer, and p-values for interaction were calculated. All statistical analyses were performed in R version 4.4.2, and two-sided p-values < 0.05 were considered statistically significant.

## Results

### Baseline characteristics of the participant

A total of 6168 participants met the inclusion criteria, of whom 506 were diagnosed with Osteoarthritis (OA) (prevalence, 7.82%) and 303 with Rheumatoid Arthritis (RA) (prevalence, 4.91%). Significant differences (p < 0.05) between OA and non-OA participants were observed for sex, age, race, marital status, PIR, BMI, smoking status, hypertension, diabetes, cancer, CRP, Triglycerides (TG), HDL, and Total Cholesterol (TC) (Table 1). Similarly, RA and non-RA participants differed significantly (p < 0.05) in age, education level, marital status, PIR, BMI, smoking status, alcohol consumption, hypertension, diabetes, cancer, CRP, and uric acid (Table 2).

**Table 1:** Population characteristics of osteoarthritis.

Characters	Non-OA (n = 5662)	OA (n = 506)	p-value
Age (years)	42.65 ± 13.80	56.36 ± 9.60	<0.001
Sex			<0.001
Female	2828 (49.95%)	323 (63.83%)	
Male	2834 (50.05%)	183 (36.17h%)	
Race			<0.001
Non-Hispanic White	2197 (38.80%)	284 (56.13%)	
Non-Hispanic Black	1166 (20.59%)	92 (18.18%)	
Mexican American	1081 (19.09%)	46 (9.09%)	
Other Hispanic	643 (11.36%)	51 (10.08%)	
Other Races	575 (10.16%)	33 (6.52%)	
Education			0.092
Less than high school	512 (9.04%)	29 (5.73%)	
High school or equivalent	812 (14.34%)	65 (12.85%)	
More than high school	4338 (76.62%)	412 (81.42%)	
Marital_status			<0.001
Never married	1184 (20.91%)	36 (7.11%)	
Married/Partner	3484 (61.53%)	332 (65.61%)	
Widowed/Divorced/Separated	994 (17.56%)	137 (27.08%)	
PIR			0.006
< 1.3	1749 (30.89%)	121 (23.91%)	
1.3‒3.5	2168 (38.29%)	184 (36.37%)	
> 3.5	1745 (30.82%)	201 (39.72%)	
BMI (kg/m^2^)			0.009
< 25	1631 (28.81%)	97 (19.17%)	
25–30	1852 (32.71%)	156 (30.83%)	
≥ 30	2179 (38.48%)	253 (50.00%)	
Smoking			<0.001
Never	3170 (55.99%)	239 (47.23%)	
Former	1198 (21.16%)	156 (30.83%)	
Current	1294 (22.85%)	111 (21.94%)	
Drinking			0.860
Never	668 (11.80%)	68 (13.44%)	
Former	627 (11.07%)	59 (11.66%)	
Current	4367 (77.13%)	379 (74.90%)	
DI-GM	4.99 ± 1.73	5.22 ± 1.76	0.085
Hypertension			<0.001
No	3708 (65.49%)	210 (41.50%)	
Yes	1954 (34.51%)	296 (58.50%)	
Diabetes			<0.001
No	4766 (84.18%)	377 (74.51%)	
Yes	896 (15.82%)	129 (25.49%)	
Cancer			<0.001
No	5363 (94.72%)	446 (88.14%)	
Yes	299 (5.28%)	60 (11.86%)	
Laboratory parameters			
CRP (mg/L)	3.73 ± 6.72	4.15 ± 7.84	0.002
Low-Density Lipoproteins (mmoL/L)	2.97 ± 0.89	3.01 ± 0.96	0.587
Triglyceride (mmoL/L)	1.29 ± 0.74	1.41 ± 0.78	0.002
Direct HDL-Cholesterol (mmoL/L)	1.40 ± 0.42	1.48 ± 0.55	0.019
Total Cholesterol (mmoL/L)	4.96 ± 1.02	5.14 ± 1.12	0.004
Creatinine (µmoL/L)	0.85 ± 0.33	0.85 ± 0.21	0.889
Uric acid (µmoL/L)	322.99 ± 80.63	323.56 ± 81.95	0.949

DI-GM, Dietary Index for Gut Microbiota; PIR, Poverty Income Ratio; BMI, Body Mass Index; CRP, C-Reactive Protein. Continuous variables were expressed as weighted means and standard deviations, while categorical variables were expressed as weighted percentages. For continuous variables, the p-value was based on the analysis of variance (ANOVA), and for categorical variables, the p-value was based on the Chi-Square test.

**Table 2:** Population characteristics of rheumatoid arthritis.

Characters	Non-RA (n = 5865)	RA (n = 303)	p-value
Age (years)	43.58 ± 14.03	53.44 ± 10.72	<0.001
Sex			0.645
Female	2984 (50.88%)	167 (55.12%)	
Male	2881 (49.12%)	136 (44.88%)	
Race			0.096
Non-Hispanic White	2377 (40.53%)	104 (34.32%)	
Non-Hispanic Black	1162 (19.81%)	96 (31.69%)	
Mexican American	1071 (18.26%)	56 (18.48%)	
Other Hispanic	662 (11.29%)	32 (10.56%)	
Other Races	593 (10.11%)	15 (4.95%)	
Education			0.001
Less than high school	503 (8.57%)	39 (12.87%)	
High school or equivalent	815 (13.90%)	61 (20.13%)	
More than high school	4547 (77.53%)	203 (67.00%)	
Marital_status			<0.001
Never married	1187 (20.25%)	33 (10.89%)	
Married/Partner	3639 (62.05%)	177 (58.42%)	
Widowed/Divorced/Separated	1038 (17.70%)	93 (30.69%)	
PIR			0.049
< 1.3	1753 (29.89%)	117 (38.61%)	
1.3‒3.5	2248 (38.33%)	104 (34.33%)	
> 3.5	1864 (31.78%)	82 (27.06%)	
BMI (kg/m^2^)			0.006
< 25	1672 (28.51%)	56 (18.48%)	
25–30	1933 (32.96%)	75 (24.75%)	
≥ 30	2260 (38.53%)	172 (56.77%)	
Smoking			0.002
Never	3289 (56.08%)	120 (39.61%)	
Former	1259 (21.47%)	95 (31.35%)	
Current	1317 (22.45%)	88 (29.04%)	
Drinking			0.002
Never	701 (11.95%)	35 (11.55%)	
Former	636 (10.84%)	50 (16.50%)	
Current	4528 (77.21%)	218 (71.95%)	
DI-GM	5.02 ± 1.73	4.77 ± 1.73	0.115
Hypertension			<0.001
No	3800 (64.79%)	118 (38.94%)	
Yes	2065 (35.21%)	185 (61.06%)	
Diabetes			<0.001
No	4932 (84.09%)	211 (69.64%)	
Yes	933 (15.91%)	92 (30.36%)	
Cancer			0.003
No	5539 (94.44%)	270 (89.11%)	
Yes	326 (5.56%)	33 (10.89%)	
Laboratory parameters			
CRP (mg/L)	3.70 ± 6.80	5.70 ± 7.46	<0.001
Low-Density Lipoproteins (mmoL/L)	2.97 ± 0.89	3.02 ± 0.98	0.743
Triglyceride (mmoL/L)	1.30 ± 0.75	1.40 ± 0.71	0.069
Direct HDL-Cholesterol (mmoL/L)	1.41 ± 0.43	1.38 ± 0.42	0.404
Total Cholesterol (mmoL/L)	4.97 ± 1.03	5.04 ± 1.06	0.587
Creatinine (µmoL/L)	0.85 ± 0.31	0.93 ± 0.62	0.057
Uric acid (µmoL/L)	322.23 ± 79.70	344.40 ± 102.61	0.016

DI-GM, Dietary Index for Gut Microbiota; PIR, Poverty Income Ratio; BMI, Body Mass Index; CRP, C-Reactive Protein. Continuous variables were expressed as weighted means and standard deviations, while categorical variables were expressed as weighted percentages. For continuous variables, the p-value was based on the analysis of variance (ANOVA), and for categorical variables, the p-value was based on the Chi-Square test.

To assess potential selection bias from missing data, the authors compared the baseline characteristics of participants included in the final analysis with those excluded due to missing covariates (Supplementary Table S2). Although statistically significant differences (p < 0.05) were found for age, race, marital status, PIR, smoking, and drinking status, the absolute differences were small. More importantly, there were no significant differences in gender distribution (p = 0.455), educational attainment (p = 0.690), BMI distribution (p = 0.337), or, most critically, the core exposure variable ‒ DI-GM score (4.89 ± 1.69 vs. 4.88 ± 1.71, p = 0.582). These findings suggest that the missing data had a limited influence on the distribution of the key exposure variable.

### Association between DI-GM and arthritis

The authors performed multivariable logistic regression to assess the association between DI-GM and arthritis. The results showed no significant association between DI-GM and Osteoarthritis (OA) in any of the three models (p > 0.05), compared with participants without arthritis. By contrast, DI-GM was significantly inversely associated with rheumatoid arthritis (RA) across all models: Model 1, OR = 0.74 (95% CI 0.57–0.97), p = 0.026; Model 2, OR = 0.64 (95% CI 0.49–0.83), p = 0.001; Model 3, OR = 0.74 (95% CI 0.56–0.98), p = 0.027. DI-GM scores were categorized into four groups. In the fully adjusted model, participants in the highest category (scores 6–12) had a significantly lower risk of RA than those in the lowest category (scores 0–3) (OR = 0.74, 95% CI 0.56–0.98, p = 0.041) (Table 3).

**Table 3:** Associations between osteoarthritis/rheumatoid arthritis and DI-GM.

Variables	Outcome	Model 1	Model 2	Model 3
		OR (95% CI)	OR (95% CI)	OR (95% CI)
		p-value	p-value	p-value
OA	DI-GM	1.27 (0.98, 1.65)	0.93 (0.73, 1.19)	0.97 (0.76, 1.24)
		0.081	0.548	0.806
	DI-GM group			
	0‒3	1.00 (Reference)	1.00 (Reference)	1.00 (Reference)
	4	1.08 (0.92, 1.26)	0.93 (0.78, 1.11)	0.98 (0.81, 1.18)
		0.326	0.454	0.854
	5	1.10 (0.89, 1.36)	0.86 (0.68, 1.08)	0.92 (0.73, 1.16)
		0.358	0.209	0.504
	6‒12	1.42 (1.19, 1.69)	0.92 (0.76, 1.12)	1.07 (0.87, 1.31)
		<0.0001	0.422	0.489
	P for trend	0.001	0.305	0.690
RA	DI-GM	0.74 (0.57, 0.97)	0.64 (0.49, 0.83)	0.74(0.56, 0.98)
		0.026	0.001	0.027
	DI-GM group			
	0‒3	1.00 (Reference)	1.00 (Reference)	1.00 (Reference)
	4	0.86 (0.69, 1.07)	0.81 (0.64, 1.03)	0.88 (0.69, 1.11)
		0.187	0.090	0.285
	5	0.82 (0.64, 1.04)	0.73 (0.57, 0.94)	0.81 (0.63, 1.06)
		0.115	0.018	0.128
	6‒12	0.77 (0.59, 0.99)	0.61 (0.47, 0.80)	0.74 (0.56, 0.98)
		0.044	0.001	0.041
	P for trend	0.038	<0.0001	0.044

Model 1 no covariates were adjusted; Model 2 Age, Sex, and Race were adjusted; Model 3 Age, Sex, Race, Marital_status, Education, PIR, BMI, Smoking, Drinking, Hypertension, Diabetes, Cancer, CRP, Low-Density Lipoproteins, Triglyceride, Direct HDL-Cholesterol, Total Cholesterol, Total Cholesterol, Creatinine, Uric acid were adjusted. OA, Osteoarthritis Arthritis; DA, Degenerative Arthritis; RA, Rheumatoid Arthritis.

### Regression cubic splines

**Fig. 2:**
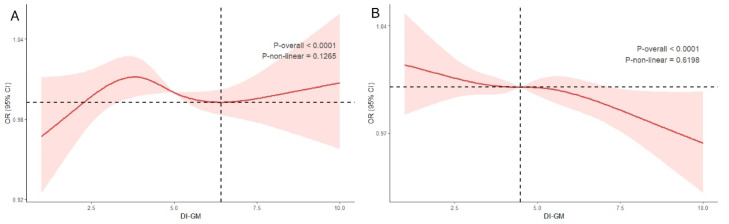
(A) Potential nonlinear relationship between DI-GM and OA; (B) Potential nonlinear relationship between DI-GM and RA. RCS regression was adjusted for age, race, marital_status, education, BMI, PIR, smoking, drinking, hypertension, diabetes, cancer, CRP, LDL, TR, HDL, TC, CR, and uric acid.

After adjusting for all covariates, the Restricted Cubic Spline (RCS) analysis showed significant overall associations but no evidence of nonlinearity between DI-GM and either Osteoarthritis (OA) or Rheumatoid Arthritis (RA) (OA: overall p < 0.0007, p for nonlinearity = 0.1265; RA: overall p < 0.0001, p for nonlinearity = 0.6198) (Fig. 2).

### Subgroup analysis

**Fig. 3:**
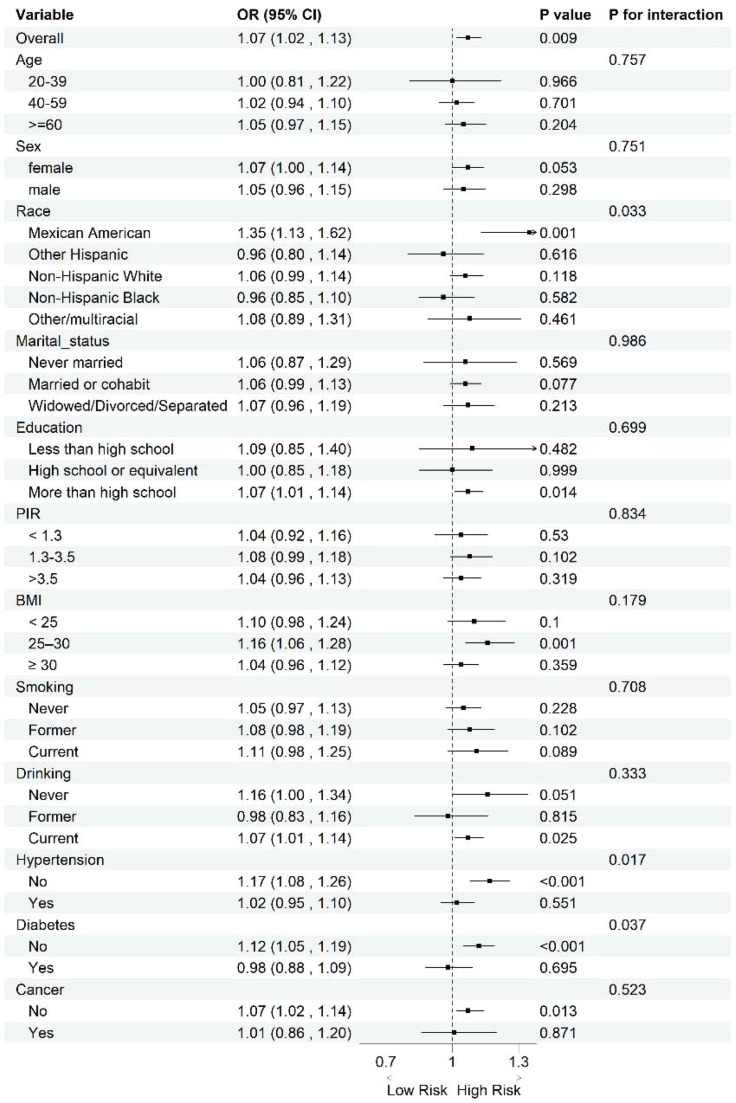
Subgroup analysis of the relationship between DI-GM and OA. Analyses were stratified for age (20‒39 years, 40‒59 years and ≥ 60-years), sex (female and male), race (Mexican American, non-Hispanic White, non-Hispanic Black, other Hispanic, and other races), education (less than high school, high school or equivalent, and more than high school), marital status (never married, married/partner, and widowed/divorced/separated), PIR (< 1.3, 1.3–3.5, and > 3.5), BMI (< 25, 25–30, and ≥ 30), smoking (never, former, and current), drinking (never, former, and current), hypertension (yes and no), diabetes (yes and no), and cancer (yes and no). These analyses are exploratory and were not adjusted for multiple comparisons; findings should be interpreted as hypothesis-generating.

**Fig. 4:**
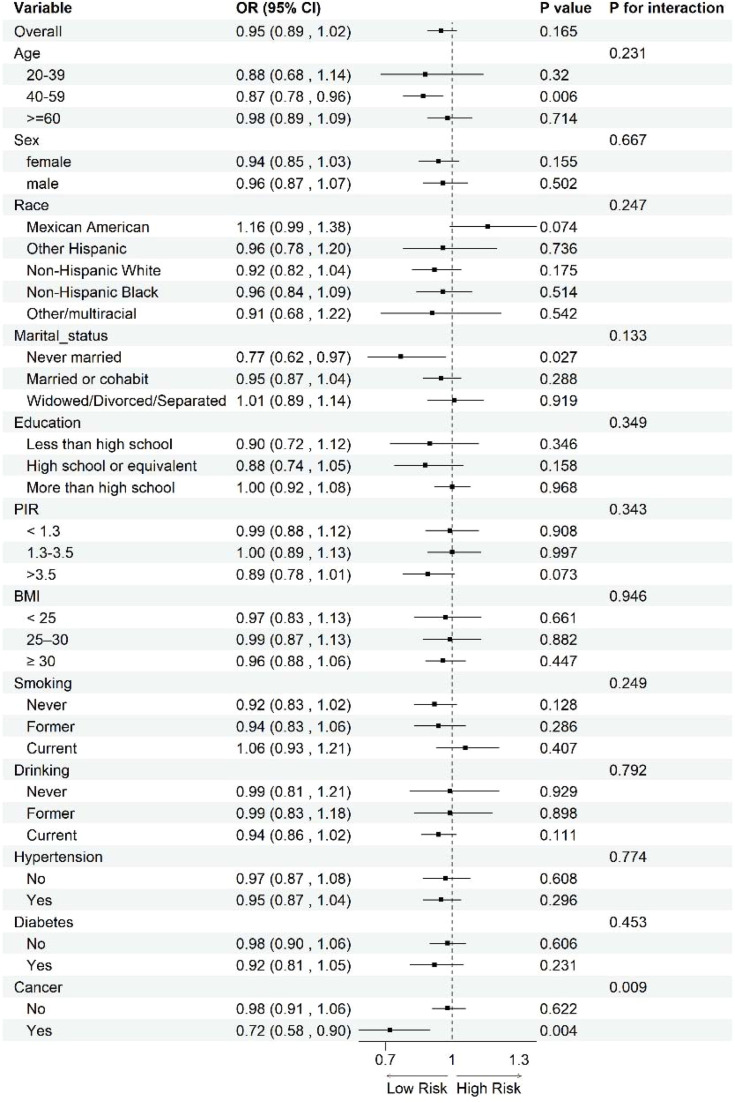
Subgroup analysis of the relationship between DI-GM and RA. Analyses were stratified for age (20‒39 years, 40‒59 years and ≥ 60-years), sex (female and male), race (Mexican American, non-Hispanic White, non-Hispanic Black, other Hispanic, and other races), education (less than high school, high school or equivalent, and more than high school), marital status (never married, married/partner, and widowed/divorced/separated), PIR (< 1.3, 1.3–3.5, and > 3.5), BMI (< 25, 25–30, and ≥ 30), smoking (never, former, and current), drinking (never, former, and current), hypertension (yes and no), diabetes (yes and no), and cancer (yes and no). These analyses are exploratory and were not adjusted for multiple comparisons; findings should be interpreted as hypothesis-generating.

It should be noted that these subgroup analyses are exploratory and require cautious interpretation due to the lack of adjustment for multiple comparisons. Exploratory subgroup analyses were performed by categorical participant characteristics without correction for multiple testing. For Osteoarthritis (OA), significant associations with DI-GM were observed among Mexican Americans, participants with more than a high school education, those with BMI 25–30 kg/m^2^, current drinkers, and individuals without hypertension, diabetes, or cancer (Fig. 3). Interaction tests indicated significant effect modification by race, diabetes, and hypertension (p < 0.05). For Rheumatoid Arthritis (RA), significant associations were found among participants aged 40–59, those who had never married, and individuals with a history of cancer (Fig. 4). A significant interaction by cancer was also observed (p < 0.05).

### Sensitivity analysis

To assess robustness, the authors performed a sensitivity analysis using Firth’s penalized likelihood method (Supplementary Table S3). The results confirmed the primary findings: the protective association between DI-GM and RA remained statistically significant across all models (for example, Model 3: OR = 0.95, 95% CI 0.92–0.99, p = 0.029), whereas no significant association was observed with OA after adjustment for confounders (Model 3: OR = 0.99, p = 0.962). These results indicate that outcome imbalance did not materially affect these conclusions.

## Discussion

Using logistic regression on NHANES 2007–2018 data, this study identified a robust inverse association between DI-GM and the risk of rheumatoid arthritis. Notably, the association was strongest in Model 2, which adjusted for demographic and socioeconomic factors (OR = 0.64), but was attenuated after further adjustment for BMI, lifestyle factors, and comorbidities in Model 3 (OR = 0.74). This pattern suggests that some of the variables added in Model 3, such as BMI, smoking, and metabolic comorbidities (e.g., hypertension, diabetes), may not be pure confounders but could lie on the causal pathway linking diet, the gut microbiome, and RA.^21^^,^^22^ Specifically, a microbiome-supportive diet might first act by influencing body weight and metabolic health, and these improvements could in turn contribute to reduced RA risk.^23^^,^^24^ Thus, Model 2 likely approximates the “total effect” of DI-GM on RA (including both direct and indirect effects via these intermediate factors), whereas the attenuated association in Model 3 more closely reflects the “direct effect” of DI-GM on RA, possibly through pathways less dependent on BMI and comorbidities, such as by directly modulating intestinal barrier integrity or systemic immune responses.^22^ Subgroup analyses further showed that the inverse association between DI-GM and RA remained significant among participants aged 40–59, among unmarried individuals, and among those with a history of cancer, lending additional support for the robustness of this relationship across different populations.

In contrast to the present findings for RA, the primary analyses detected no significant association between DI-GM and Osteoarthritis (OA). This null result may reflect outcome misclassification for OA or potential reverse causation. Notably, although the overall association was null, exploratory subgroup analyses suggested possible associations in certain groups, including Mexican Americans and overweight participants. These signals should be interpreted with extreme caution, as they are purely hypothesis-generating and require independent replication. First, the extensive subgroup testing was not corrected for multiple comparisons, which increases the risk of false positives. Second, they may point to true effect modification. For overweight individuals, obesity is often accompanied by a pro-inflammatory gut microbiota and metabolic endotoxemia. A higher DI-GM score, indicative of a diet rich in fiber and fermented foods, might mitigate obesity-related systemic inflammation and metabolic dysfunction, thereby modifying OA risk specifically in this susceptible subgroup. For Mexican Americans, unique genetic backgrounds, dietary habits (e.g., higher consumption of corn-based products or specific spices), or sociocultural factors could interact with the diet-microbiota axis, making the association between DI-GM and OA more detectable within this population. Critically, these isolated subgroup findings do not constitute robust evidence for an association between DI-GM and OA in the overall population. Therefore, the current data do not support DI-GM as a universal risk factor for OA, but these heterogeneous findings provide valuable hypothesis-generating leads and underscore the complexity of the diet to microbiome to joint axis in OA.

Currently, few studies have examined the association between the Dietary Index of the Gut Microbiome (DI-GM) and arthritis. Prior research has mainly focused on the effects of the gut microbiota and their metabolites on arthritis progression. To our knowledge, this is the first study to examine the association between DI-GM and Osteoarthritis (OA) or Rheumatoid Arthritis (RA). A 16S rRNA sequencing study of patients with RA found that, compared with healthy controls, RA patients had significantly reduced microbial richness and altered gut microbiota composition.^25^ Multiple studies have reported an increased abundance of Prevotella copri and decreased Bacteroides species in the early stages of RA.^26^^,^^27^ Scher et al. observed significant enrichment of Prevotellaceae in the gut microbiota of RA patients, suggesting that the P.copri genome may serve as a biomarker of RA-associated microbiota.^28^ Kishikawa et al., using metagenomic analysis, further reported that several Prevotella species, in addition to P.copri, were enriched in the RA gut microbiome.^29^ Another study evaluating gut microbiota in rheumatic diseases found that RA patients had reduced numbers of butyrate-producing (anti-inflammatory) bacteria and enrichment of proinflammatory taxa.^30^ Moreover, several animal studies have reported significantly reduced gut microbiota diversity in collagen-induced arthritis mouse models.^31^, ^32^, ^33^

The association between the gut microbiota and Rheumatoid Arthritis (RA) risk involves multiple mechanisms, including molecular mimicry, impaired intestinal barrier function, immune responses induced by gut microbes and their metabolites, and the contribution of Human Leukocyte Antigen (HLA) alleles.^34^ Studies have found that Prevotella proteins share high sequence homology with N‑acetylglucosamine‑6‑sulfatase (GNS), and citrullinated GNS may drive T and B‑cell-mediated autoimmunity in RA.^35^ The intestinal epithelial barrier plays a crucial role in pathogen defense and immune homeostasis.^36^ In RA patients, barrier integrity is compromised, with increased permeability that may allow mucosal immune cells to traffic from the gut to the joints and contribute to cartilage damage.^37^ A recent study reported that P. copri can stimulate T‑cell-mediated production of IgA and IgG antibodies, influencing RA progression.^38^ Additionally, Lipopolysaccharide (LPS) released by commensal Gram‑negative bacteria binds Toll‑Like Receptor 4 (TLR4) on macrophages and synovial cells, triggering a robust cytokine response that promotes synovitis and cartilage destruction.^34^ Genetic studies have identified certain HLA alleles as major RA susceptibility factors; carriers are more likely to produce Anti‑Citrullinated Protein Antibodies (ACPAs).^39^ Collectively, these findings support the role of diet in relation to RA. A low DI‑GM score ‒ reflecting diets high in red/processed meat and low in fiber ‒ is associated with systemic inflammation and metabolic dysregulation, which are known contributors to RA pathogenesis. While this dietary pattern is hypothesized to act via gut dysbiosis (e.g., increased Prevotella abundance and enhanced ACPA production), the study design cannot confirm such mediating pathways. The present study provides evidence supporting further investigation of the diet-RA relationship, including the role of the gut microbiota.

This study has several limitations. First, arthritis status was based on self-reported physician diagnosis, which may introduce misclassification bias. For rheumatoid arthritis, no serologic confirmation was available; for osteoarthritis, diagnostic agreement with imaging is modest, so the control group may include undiagnosed cases. Such nondifferential misclassification would likely attenuate true associations and may partly explain the null findings for OA. Second, the cross-sectional design precludes causal inference and cannot fully rule out confounding from factors such as healthcare-seeking behavior. Third, DI-GM is a dietary proxy derived from 24-hour dietary recalls rather than a direct measure of the gut microbiome, and therefore may be affected by recall bias and day-to-day variation in intake. Finally, the high rate of participant exclusion (> 75%) due to missing data raises concerns about selection bias. While key characteristics, including the exposure (DI-GM score), were comparable between included and excluded groups (Supplementary Table S2), the excluded participants differed in some sociodemographic factors. This may limit the generalizability of these findings, and the results should be interpreted primarily within the context of the included sample.

Despite these limitations, the study has several important strengths. First, it is the first investigation of the association between the Dietary Index of the Gut Microbiome (DI-GM) and distinct types of arthritis. Second, the complex multistage sampling design and the pooling of multiple NHANES cycles support the national representativeness and robustness of the findings. The present results provide new insights into the relationship among diet, the gut microbiome, and joint health, highlight the potential of diet-based modulation of the gut microbiome for the prevention of rheumatoid arthritis, and underscore the need for more precise case definitions in future osteoarthritis research.

## Conclusion

In conclusion, this cross-sectional study found an inverse association between the Dietary Index (DI-GM) and rheumatoid arthritis. In this cross-sectional design, the authors cannot distinguish whether a higher DI-GM score is inversely associated with RA or whether an RA diagnosis leads individuals to adopt a diet that scores higher on the DI-GM (e.g., more anti-inflammatory foods, less red meat). This reverse causality is a fundamental limitation that precludes any causal inference. Therefore, these findings are best interpreted as generating a hypothesis that diet, potentially via the gut microbiome, may be related to RA. Future prospective cohort studies are essential to establish the temporal sequence, clarify the direction of any association, and control for potential reverse causation. Such studies are also needed to elucidate the underlying microbial mechanisms.

## Ethics approval

This study was performed in line with the principles of the STROBE Statement. The NHANES study procedures were reviewed and approved by the NCHS Ethics Review Board. Informed consent was obtained from all subjects involved in NHANES (Protocol #2005–06 和 Protocol #2011–17).

## Author’s contributions

Weihua Yang, Zhi Li, Xiang Chen, Jinian Chen, Zhiming Lu participated in the data collection. Weihua Yang and Zhi Li wrote the manuscript. Xiang Chen and Jinian Chen analyzed the data. Zhiming Lu reviewed and edited the manuscript. All authors read and approved the final manuscript.

## Funding

The authors declare that no financial support was received for the research, authorship, and/or publication of this article.

## Declaration of competing interest

The authors declare no conflicts of interest.

## Data Availability

The datasets presented in this study can be found in online repositories (https://www.cdc.gov/nchs/nhanes/index.htm).

## Appendix Supplementary materials

Image, application 1

## Footnotes

Supplementary material associated with this article can be found, in the online version, at doi:10.1016/j.clinsp.2026.100995.
