# Supplementary material for: Association between a gut microbiota-targeted dietary index and osteoarthritis/rheumatoid arthritis: a cross-sectional study
Source: Clinics (Sao Paulo). 2026 May 19;81:100995. doi: 10.1016/j.clinsp.2026.100995 (PMC13213782; doi:10.1016/j.clinsp.2026.100995)
Supplement: Supplementary file 1 [file mmc1.docx]

**CLINICS-D-25-01290_Supplementary Material**

**Table S1** Components of the DI-GM.

| **Component** | **Included Foods within the Component** | **Scoring** |
| --- | --- | --- |
| Beneficial to gut microbiota |  |  |
| Avocados | Avocados | For each component, a score of 1 if consumption at or above the sex-specific median, else 0 |
| Broccoli | Broccoli |  |
| Chickpea | Chickpeas |  |
| Coffee | Coffee |  |
| Cranberries | Cranberries |  |
| Fermented dairy | Yogurt, cheese, kefir, sour cream, buttermilk |  |
| Fiber | Not applicable |  |
| Green tea | Green tea |  |
| Soybean | Soy products ‒ Soy milk, Tofu |  |
| Whole grains | Grains defined as whole grains, containing the entire grain kernel ‒ the bran, germ, and endosperm |  |
| Unfavorable to gut microbiota |  |  |
| High-fat diet (% energy) | Not applicable | 0 if consumption at or above 40% energy from fat, else 1 for each remaining component, a score of 0 if consumption at or above the sex-specific median, else 1 |
| Processed meat | Frankfurters, sausages, corned beef, and luncheon meat that are made from beef, pork, or poultry |  |
| Red meat | Beef, veal, pork, lamb, and game meat; excludes organ meat and cured meat |  |
| Refined grains | Refined grains that do not contain all of the components of the entire grain kernel |  |

**Table S2** Associations between osteoarthritis/rheumatoid arthritis and DI-GM: firth’s regression analysis.

| **Variables** | **Model 1** | **Model 2** | **Model 3** |
| --- | --- | --- | --- |
|  | **OR (95% CI)** | **OR (95% CI)** | **OR (95% CI)** |
|  | **p** | **p** | **p** |
| Non-arthritis | Reference | Reference | Reference |
| OA | 1.07 (1.01, 1.12) | 0.98 (0.92, 1.03) | 0.99 (0.94, 1.05) |
|  | 0.012 | 0.484 | 0.962 |
| Non-arthritis | Reference | Reference | Reference |
| RA | 0.94 (0.91, 0.98) | 0.92 (0.88, 0.95) | 0.95 (0.92, 0.99) |
|  | 0.003 | <0.001 | 0.029 |

Model 1 no covariates were adjusted; Model 2 Age, Sex, and Race were adjusted; Model 3 Age, Sex, Race, Marital_status, Education, PIR, BMI, Smoking, Drinking, Hypertension, Diabetes, Cancer, CRP, Low-Density Lipoproteins, Triglyceride, Direct HDL-Cholesterol, Total Cholesterol, Total Cholesterol, Creatinine, Uric acid were adjusted. OA, Osteoarthritis Arthritis; DA, Degenerative Arthritis; RA, Rheumatoid Arthritis.

**Table S3** Comparison of baseline characteristics between included and excluded participants.

| **Characters** | **Excluded (n = 19,426)** | **Included (n = 6,168)** | **p-value** |
| --- | --- | --- | --- |
| Age (years) | 44.26 ± 14.29 | 44.78 ± 14.34 | 0.013 |
| Sex |  |  |  |
| Female | 9394 (48.35%) | 3017 (48.91%) | 0.455 |
| Male | 10032 (51.65%) | 3151 (51.09%) |  |
| Race |  |  |  |
| Non-Hispanic White | 7110 (36.60%) | 2481 (40.22%) | <0.001 |
| Non-Hispanic Black | 4537 (23.36%) | 1258 (20.40%) |  |
| Mexican American | 3078 (15.84%) | 1127 (18.27%) |  |
| Other Hispanic | 2141 (11.02%) | 694 (11.25%) |  |
| Other Races | 2560 (13.18%) | 608 (9.86%) |  |
| Education |  |  |  |
| Less than high school | 1745 (8.99%) | 541 (8.77%) | 0.690 |
| High school or equivalent | 2684 (13.83%) | 877 (14.22%) |  |
| More than high school | 14980 (77.18%) | 4750 (77.01%) |  |
| Marital_status |  |  |  |
| Never married | 4279 (22.03%) | 1220 (19.78%) | 0.001 |
| Married/Partner | 11622 (59.83%) | 3816 (61.87%) |  |
| Widowed/Divorced/Separated | 3525 (18.14) | 1132 (18.35%) |  |
| PIR |  |  |  |
| < 1.3 | 5825 (33.90%) | 1870 (30.32%) | <0.001 |
| 1.3‒3.5 | 6071 (35.34%) | 2352 (38.13%) |  |
| > 3.5 | 5285 (30.76%) | 1946 (31.55%) |  |
| BMI (kg/m^2^) |  |  |  |
| < 25 | 5520 (28.69%) | 1728 (28.02%) | 0.337 |
| 25–30 | 6080 (31.60%) | 2008 (32.55%) |  |
| ≥ 30 | 7640 (39.71%) | 2432 (39.43%) |  |
| Smoking |  |  |  |
| Never | 11086 (57.07%) | 3409 (55.27%) | 0.003 |
| Former | 3885 (20.00%) | 1354 (21.95%) |  |
| Current | 4455 (22.93%) | 1405 (22.78%) |  |
| Drinking |  |  |  |
| Never | 2334 (13.56%) | 736 (11.93%) | 0.005 |
| Former | 1925 (11.18%) | 686 (11.12%) |  |
| Current | 12965 (75.27%) | 4746 (76.95%) |  |
| DI-GM | 4.89 ± 1.69 | 4.88 ± 1.71 | 0.582 |
| Hypertension |  |  |  |
| No | 11829 (62.51%) | 3918 (63.52%) | 0.159 |
| Yes | 7094 (37.49%) | 2250 (36.48%) |  |
| Diabetes |  |  |  |
| No | 16534 (85.11%) | 5143 (83.38%) | 0.001 |
| Yes | 2892 (14.89%) | 1025 (16.62%) |  |
| Cancer |  |  |  |
| No | 18195 (93.76%) | 5809 (94.18%) | 0.243 |
| Yes | 1211 (6.24%) | 359 (5.82%) |  |

DI-GM, Dietary Index for Gut Microbiota; PIR, Poverty Income Ratio; BMI, Body Mass Index. Continuous variables were expressed as weighted means and standard errors, while categorical variables were expressed as weighted percentages. For continuous variables, the p-value was based on the Analysis of Variance (ANOVA), and for categorical variables, the p-value was based on the Chi-Square test.
